# A novel surgical model for the preclinical assessment of the osseointegration of dental implants: a surgical protocol and pilot study results

**DOI:** 10.1186/s12967-021-02944-w

**Published:** 2021-06-28

**Authors:** Noura M. AlOtaibi, Michael Dunne, Ashraf F. Ayoub, Kurt B. Naudi

**Affiliations:** 1 Department of Oral and Maxillofacial Surgery, Glasgow University Dental Hospital and School, 378 Sauchiehall Street, Glasgow, G23JZ UK; 2 Oral and Maxillofacial Surgery, King Saud University, Riyadh, 11362 Saudi Arabia; 3 Institute of Cardiovascular and Medical Sciences, University of Glasgow, Glasgow, G12 8QQ UK

**Keywords:** Surgical model, Osseointegration, In vivo study, Dental implants, Preclinical investigation

## Abstract

**Background:**

Dental implants are considered the gold standard replacement for missing natural teeth. The successful clinical performance of dental implants is due to their ability to osseointegrate with the surrounding bone. Most dental implants are manufactured from Titanium and it alloys. Titanium does however have some shortcomings so alternative materials are frequently being investigated. Effective preclinical studies are essential to transfer the innovations from the benchtop to the patients. Many preclinical studies are carried out in the extra-oral bones of small animal models to assess the osseointegration of the newly developed materials. This does not simulate the oral environment where the dental implants are subjected to several factors that influence osseointegration; therefore, they can have limited clinical value.

**Aim:**

This study aimed to develop an appropriate *in-vivo* model for dental implant research that mimic the clinical setting. The study evaluated the applicability of the new model and investigated the impact of the surgical procedure on animal welfare.

**Materials and methods:**

The model was developed in male New Zealand white rabbits. The implants were inserted in the extraction sockets of the secondary incisors in the maxilla. The model allows a split-mouth comparative analysis. The implants’ osseointegration was assessed clinically, radiographically using micro-computed tomography (µ-CT), and histologically. A randomised, controlled split-mouth design was conducted in 6 rabbits. A total of twelve implants were inserted. In each rabbit, two implants; one experimental implant on one side, and one control implant on the other side were applied. Screw-shaped implants were used with a length of 8 mm and a diameter of 2 mm.

**Results:**

All the rabbits tolerated the surgical procedure well. The osseointegration was confirmed clinically, histologically and radiographically. Quantitative assessment of bone volume and mineral density was measured in the peri-implant bone tissues. The findings suggest that the new preclinical model is excellent, facilitating a comprehensive evaluation of osseointegration of dental implants in translational research pertaining to the human application.

**Conclusion:**

The presented model proved to be safe, reproducible and required basic surgical skills to perform.

**Supplementary Information:**

The online version contains supplementary material available at 10.1186/s12967-021-02944-w.

## Clinical relevance

- **Scientific rationale for the study**: the use of in-vivo models to assess a new dental implant is essential to study osseointegration, inflammation or immunological reactions within a live model. Animal model is a crucial step for translational research and is challenging both technically and biologically. Most of in-vivo studies evaluated the oral implants in long bones, which is not relevant to clinical application,
- **Principal findings**: we successfully introduced a new model for dental implants investigation in rabbits' maxilla, which solves the issues associated with previous models,
- **Practical implications**: to explore biological efficacy of modified implants, an appropriate model is required that resembles the clinical situation before considering human applications.

## Background

The replacement of missing teeth with dental implants is considered the gold standard approach for oral rehabilitation [1]. Despite the significant success rates of the currently-used dental implants, researchers are exploring new materials and investigating the effects of surface modifications (micro-geometries) on direct bone formation around implants in the maxilla and mandible “osseointegration” [2, 3]. Several new materials have been approved for the fabrication of dental implants; however, some have been tested in preclinical models that do not completely replicate the clinical environment. Multiple large animals have been previously considered for the preclinical assessment of the osseointegration of dental implants, these include; dogs, pigs, sheep and non-human primates [4]. There are major drawbacks which limit the routine use of large animals to asses of the osseointegration of dental implants such as; expense, limited availability, handling difficulties, need for specialized housing centers and specially trained staff as well as ethical considerations related to protected species [4–7]. Moreover, there is a risk of cross-infection of tuberculosis and other zoonotic diseases [6, 8]. Small animals, in comparison, are easier to handle, less expensive to acquire and maintain and have a satisfactory bone turnover rate to study osseointegration [6, 9]. They also do not require specialized housing and are available in athymic, transgenic and knockout strains [10].

Rabbits are mammalian animals which are biologically comparable to humans [11]. The rabbit’s maxilla is similar in morphology to the human one; embryologically it develops by intramembranous ossification. Another added advantage is the fact that the rabbit is classified as a small animal and so can be housed in a small animal facility, but it is large enough to allow the testing of the osseointegration of dental implants. Rabbits are considered a reliable model for studies related to bone regeneration, periodontal wound healing and the integration of dental implants [10–16].

The available evidence suggests that osseointegration of oral implant is substantially dissimilar from osseointegration in long bones [17]. At present many preclinical investigations of dental implants are carried out in the long bones and the cranium [18–25]. These are of a limited clinical application because the oral cavity represents a challenging environment for implant osseointegration; thus, dental implants should be tested in the maxilla and mandible to investigate the impact of the saliva, oral microbiology and the biting forces on the osseointegration process [17]. Therefore, there is a need to develop a clinically valid experimental model for the testing of dental implants [9].

**Fig. 1:**
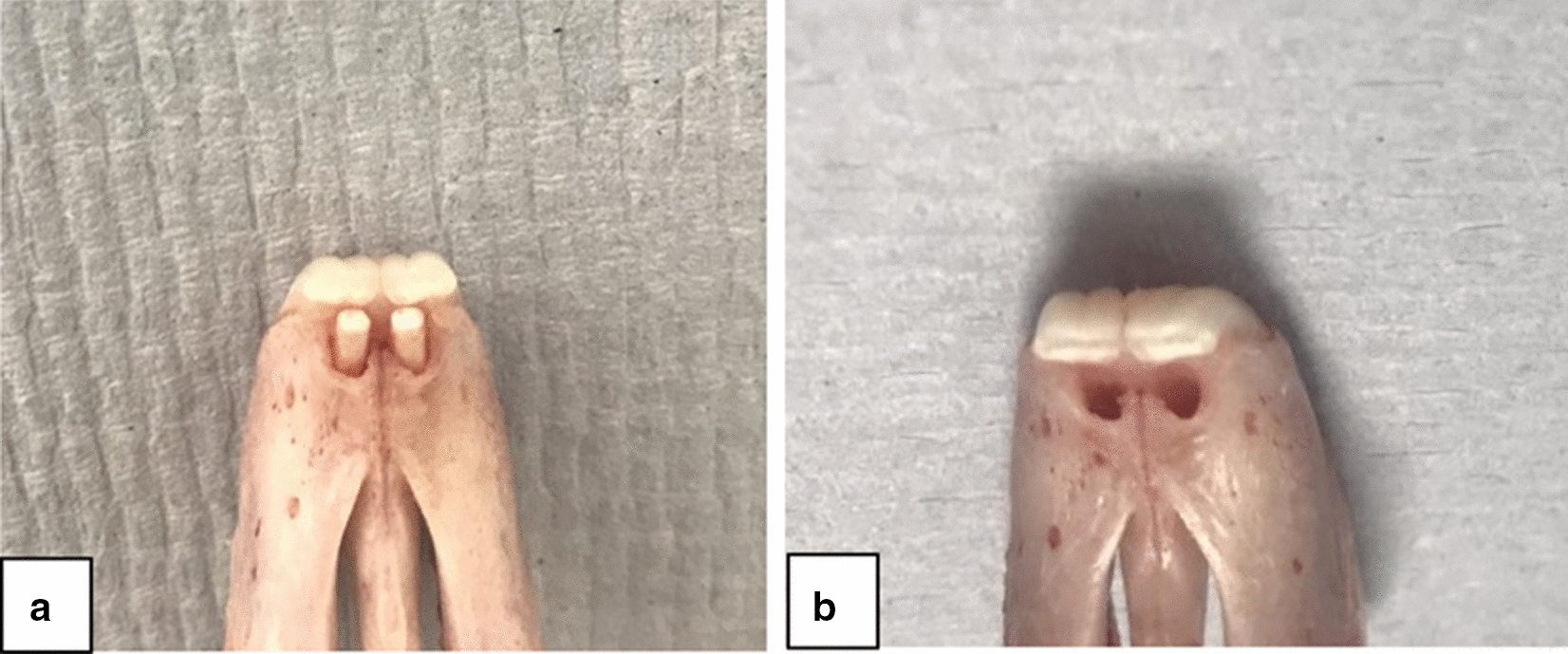
Cadaveric maxilla of rabbit. **a** Illustration of position of the secondary incisors behind the primary incisors. **b** The proposed implant positions in the sockets of the extracted secondary incisors

Our goal was to recapitulate the unique environment of implant osseointegration in the oral cavity using a rabbit model for future dental implants research. Anatomically, the two primary maxillary incisors are fully erupted on the anterior part of the rabbit maxilla, in addition, two secondary incisors are barely visible on their palatal surface and are known as peg teeth (Fig. 1). The lower incisors occlude against these secondary incisors. The main function of the secondary incisors is controlling the overeruption of the lower incisors and shaping their edges [26]. Hypothetically, the extraction of these secondary incisors will not affect the animal welfare and simultaneously subject the implants to indirect load from the lower incisors.

In the presented study, the implants were inserted in the extraction sockets of the secondary incisors in the maxilla. The model allows a split-mouth comparative analysis. In order to validate the experimental method and the sample size, the pilot study was conducted.

## Materials and methods

### Ethics approval

Approval was obtained from the Animal Welfare and Ethical Review Board of the University of Glasgow and licensed by the UK Home Office under the (Animals (Scientific Procedures Act) 1986). The *in-vivo* study was carried out at the Small Animals Research Unit-Biological Services, University of Glasgow. All animal husbandry regulations and ARRIVE guidelines for preclinical studies were followed.

### Animals

This study was carried out on 6 male New Zealand white rabbits, the weight of each one ranged from 2.8 to 3.7 kg. The rabbits, provided by Envigo, UK, were acclimatized for two weeks before surgery. Each rabbit was housed separately in a standard cage and maintained in a room with controlled temperature under 12 h light-dark cycles.

### Sample size calculation

The resource equation method was used to estimate the required number of animals [27]:

1 $${\text{E}} = {\text{total}}\,{\text{number}}\,{\text{of}}\,{\text{observations}} - {\mkern 1mu} {\text{total}}\,{\text{number}}\,{\text{of}}\,{\text{groups}}.$$

E= (12)–2 = 10, which is considered an adequate sample size according to Festing and Altman [28].

### Study design

A randomized, controlled, split-mouth study was performed. In each rabbit, two implants were inserted; an experimental implant on one side, and a control one on the other side. These were randomly assigned to the right and left extraction sockets of the secondary maxillary incisors (Fig. 1) using a computer randomization programme. A total of twelve implants were inserted in 6 rabbits. The implants were screw shaped mini-implants, 8 mm in length and 2 mm in diameter. The experimental implants were made of medical-grade polyetheretherketone material provided by Invibio Biomaterial Solutions, UK and the implants were manufactured by Ensinger, UK. The titanium implants (controls) were supplied by Cimplant Co., Seoul, Korea.

### Anaesthesia

The rabbits were anaesthetised by subcutaneous (SC) injection of Narketan (ketamine) (15 mg/kg) and Domitor (medetomidine) (0.25 mg/kg). After 10 min, an oral endotracheal tube was inserted and fixed on the right side around the mandible and the neck using a bandage. Ophthalmic lubricant Viscotears Liquid Gel was applied on both eyes. General anaesthesia was maintained using isoflurane and oxygen at a ratio of 1:1. To reverse the muscle relaxant action, Antisedan (atipamezole) (0.25 mg/kg) was injected intramuscularly (IM) and an infraorbital nerve block were administered (Additional file 1: Fig. S1). Body temperature was monitored pre- and postoperatively, as well as for three days postoperatively using a rectal digital thermometer.

### Implementation of the surgical model

The surgical technique was developed, refined, and finalized following preliminary cadaveric experimentation.

**Fig. 2:**
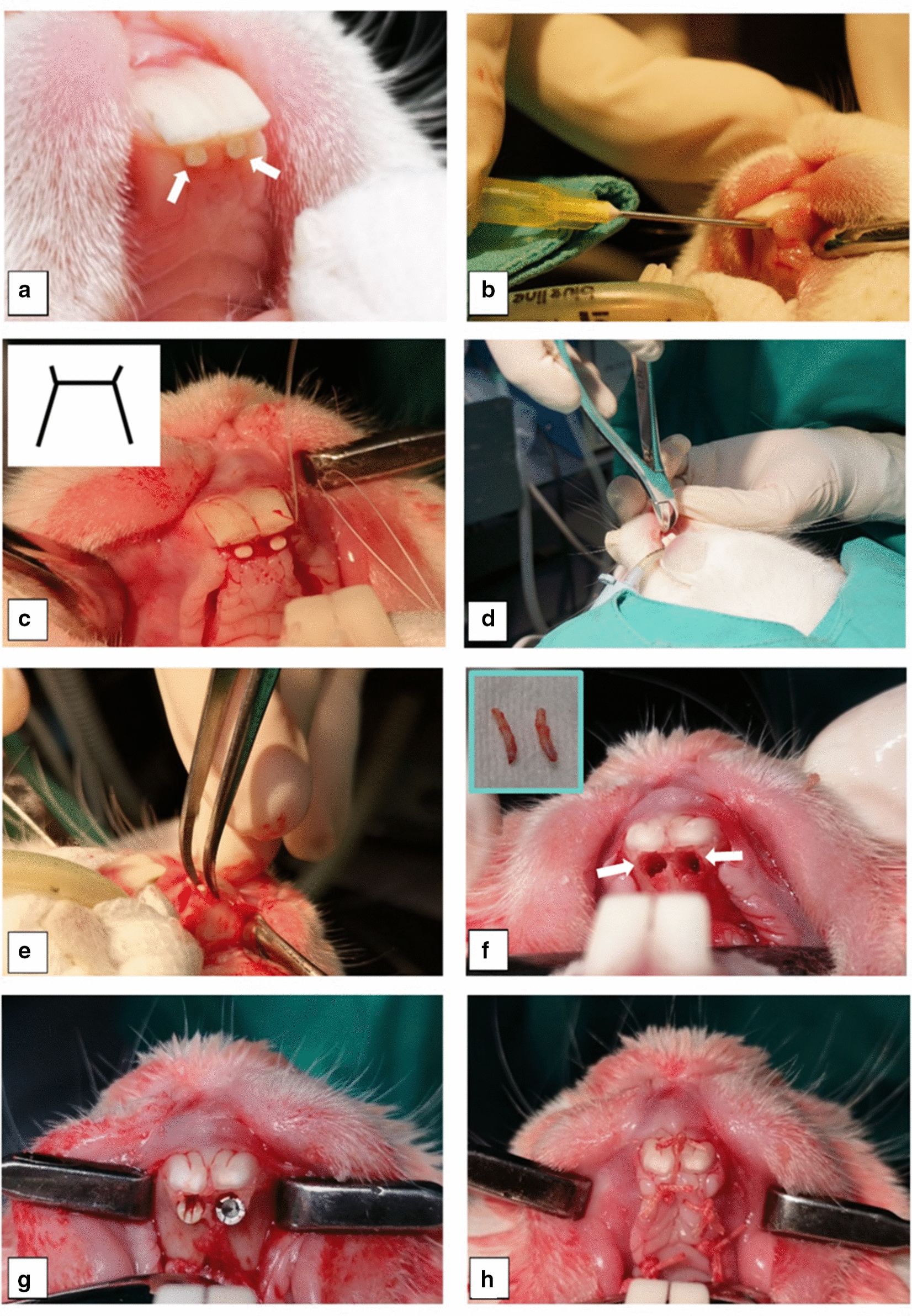
Operative protocol. **a** Secondary incisors (arrows). **b** Secondary incisors luxated with a 19-gauge. **c** H-shape flap design. **d** Extraction performed using root forceps. **e** Alternative extraction method using college tweezers. **f** Extraction sockets (arrows) and extracted secondary incisors at upper left corner. **g** Final placement of implants in the maxilla. **h** Reflected flap closed primarily using bio-absorbable sutures

A throat pack was inserted to secure the endotracheal tube in place and minimize the leakage of oral fluids. Oral disinfection was done using chlorhexidine solution Vetasept (Chlorhexidine Gluconate 0.5 % Surgical Scrub Solution Animalcare Ltd, York, UK). The surgical site was draped in the standard manner (Additional file 1: Fig. S1). Local anaesthesia was injected in the palatal mucosa using 2% Xylocaine DENTAL with epinephrine 1:80,000, (DENTSPLY Pharmaceutical, York, UK). The secondary incisors were luxated using a 19-gauge needle to sever the periodontal ligaments around the teeth and to reduce the chance of root fracture (Fig. 2a, b). A rectangular palatal flap was raised (approximately 1 cm x 0.8 cm x 1 cm) from the first premolar of one side to the contralateral first premolar by performing a full-thickness incision in the palatal mucosa with a No.15 blade, the mucoperiosteal flap was raised using a periosteal elevator to expose the bone. An H-shape flap was obtained by cutting anterior releasing incisions which were extended labially (Fig. 2c). The loosened secondary incisors were extracted using college tweezers or root forceps (Fig. 2d, e). The primary incisors were trimmed 5 mm from the incisal edge using a fissure carbide bur which facilitated the insertion of the dental implants parallel to the longitudinal axis of the secondary incisor. Fractures of the roots of the secondary incisors during the extraction was one of the challenges of the surgical technique which was managed with the delicate application of a 19-gauge needle to luxate and cut the periodontal ligament around these incisors and drilling of the remaining root fragments using a fissure bur if fracture happened.

The extraction sockets were prepared using a low-speed 1.5 mm twist drill to a depth of 8 mm under copious saline irrigation. The implants were inserted manually in the prepared sockets (Fig. 2g). Primary stability was determined clinically, the mobility was checked using a dental probe, the implants were immobile and achieved primary stability within the sockets. The muffled sound on percussion conformed the primary stability of the implants. Before suturing, the surgical site was irrigated with saline. The reflected flap was sutured primarily over the implants using bio-absorbable sutures (Vicryl Rapide suture, Ethicon) size 3–0 in an interrupted manner (Fig. 2h). The surgical procedure for each rabbit took between 20 and 30 min.

### Analgesia and postoperative care

Following surgery, the rabbits were housed in a recovery cage overnight then were returned to their standard cage the next day. They were inspected daily in the postoperative period for the assessment of behavioral changes indicating distress, and the weight was recorded. The signs of pain and discomfort postoperatively were assessed using the rabbit grimace scale, the necessary analgesia was provided following consultation with the Named Veterinary Surgeon (NVS) of the unit. Soft food was provided during the first three postoperative days followed by gradual introduction of semi-solid diets.

The rabbits were humanely euthanatized after eight weeks with an overdose of pentobarbitone sodium (140 mg/kg) according to the Schedule 1 method. The euthanasia of the animals occurred in seconds, which was confirmed by cardiac arrest and cessation of involuntary reflexes. The maxillae were explanted and the surrounding soft tissue was dissected from the bone (Fig. 5). Bone formation around the dental implants was evaluated using µ-CT followed by histological assessment.

### Micro-computed tomography and histological assessments

The anterior part of the maxilla was harvested, fixed in 10% formaldehyde solution, then dehydrated in a series of graded ethanol before resin embedding. Following fixation, the bony segments with the dental implants were imaged using micro-computed tomography (µ-CT). Micro-CT images of bone samples were obtained using a SkyScan 1072 scanner (Bruker, Germany), SHT 11 Megapixel camera and a Hamamatsu 80 kV (100 µA) source at 80 kV, 1050 msec (exposure time), 6.75 μm (resolution), 0.2° (rotation step), and 180° (rotation angle). No filter was applied to the X-Ray source. The three-dimensional regenerated bone was reconstructed from micro-CT images using the CTAn software package (Skyscan). An annular region of a thickness of 100 µm from the implant surface was considered the region of interest (ROI), parameters such as percent bone volume (BV/TV) and bone mineral density (BMD) were measured. Measurements of the bone density were based on two phantoms of calcium hydroxyapatite (CaHA) with known mass concentrations of 0.25 g/cm3 and 0.75 g/cm3 were scanned under the same scanning setting. This allowed the calibration of the attenuation of the study samples based on the linear interpolation between the two known densities. The BV/Total Volume (TV) was calculated using the formula: BV/TV (%) = bone volume in the band / total tissue volume of the selected band. After complete curing of the resin, the specimens were sectioned using a precision saw, into blocks of a thickness of 200 µm. The blocks were trimmed to 50µm for histological assessment.

### Statistical analysis

All quantitative data are expressed as mean ± standard deviations. Statistical analysis was performed using GraphPad Prism 8 software. Normal distribution was tested using Shapiro-Wilk normality test followed by two-tailed paired t-test for parametric data and Mann-Whitney U test for non-parametric data. Statistical significance was accepted at p < 0.05.

## Results

### Clinical findings

**Fig. 3:**
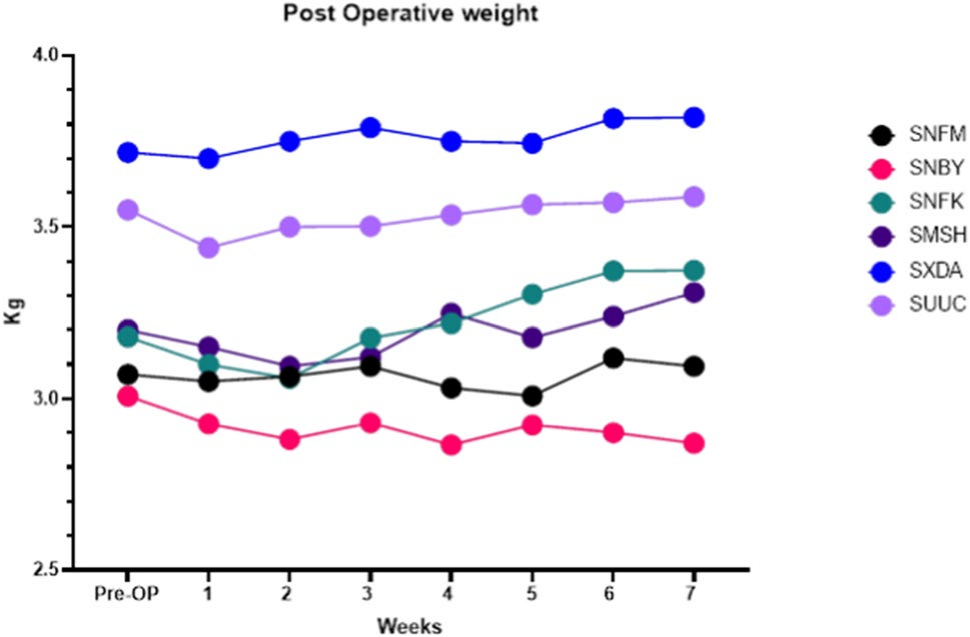
Chart of individual bodyweight of rabbits during the study period

**Fig. 4:**
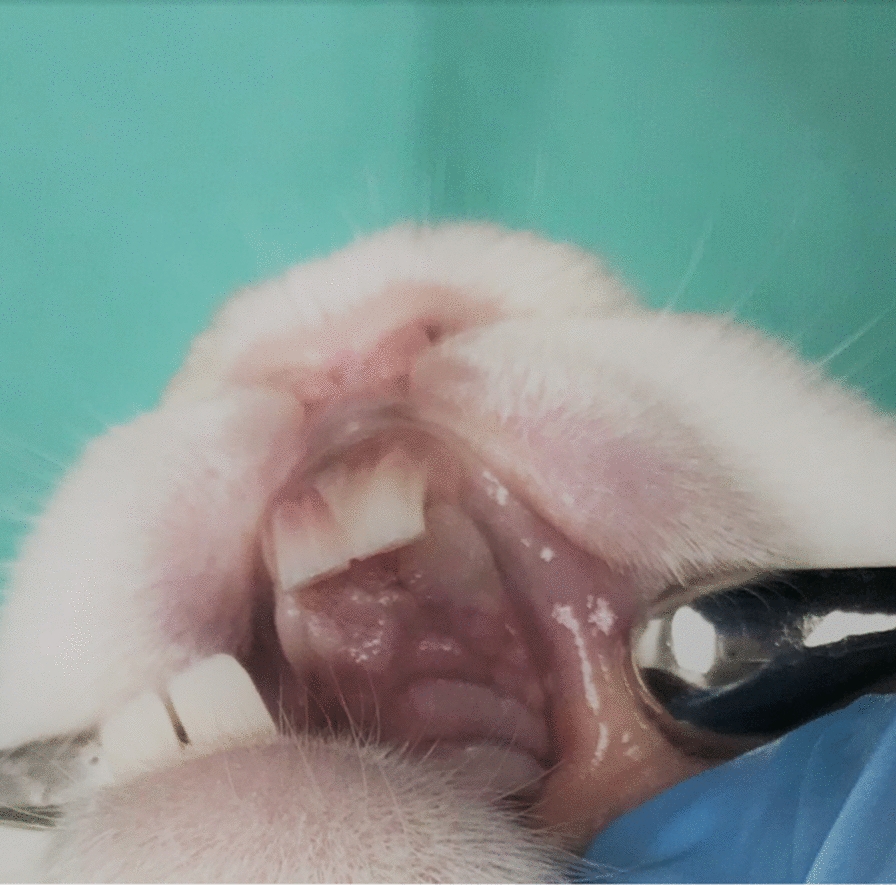
Clinical image demonstrating complete healing with healthy gingival coverage in one of the cases postoperatively

The surgical procedures were completed uneventfully in the 6 cases. All cases recovered well with minimal postoperative complications. All rabbits started to eat normally and regained ≥ 96% of their preoperative weight at one week postoperatively (Fig. 3). The weight loss was associated with pain and inability to eat. All rabbits recovered by the third week and regained their preoperative weight and behaviour. The surgical site was accessible for inspection and was regularly monitored during the healing period allowing for maintenance of oral hygiene (Fig. 4). The upper primary incisors regrew to their average length within ten days postoperatively. The implants remained submerged and covered by healthy gingiva throughout the study period. None of the cases encountered unexpected implant failure or developed wound dehiscence around the implants or any signs of infection.

### Gross examination of the retrieved bone sample and implants

**Fig. 5:**
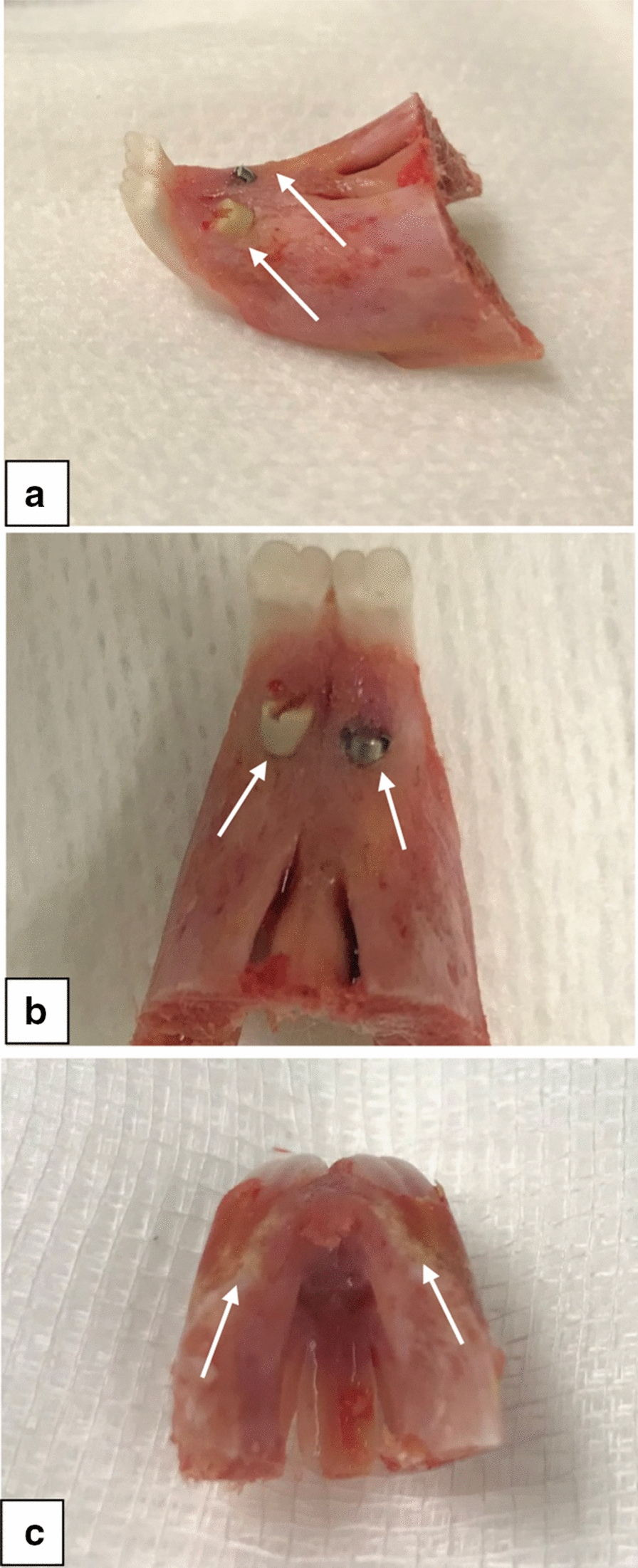
Gross examination of the retrieved bone sample. **a** Close-up of lateral view of explanted anterior maxilla of one of the cases illustrating the complete bone coverage of both implants. **b** Close-up view showing no bone overgrowth nor marginal bone resorption around both implants. **c** Superior view showing intact bone with no bone perforation by the implants

The colour and texture of the gingival tissue appeared healthy and did not reveal signs of inflammation or any adverse tissue response. In all the cases, no fibrous tissue was found around both implants (Fig. 5). Upon dissection of the soft tissues, no bony overgrowth nor marginal bone resorption was observed around the implants. All implants were stable and immobile, which provided a preliminary indication of osseointegration. The stability was tested clinically by evaluating the mobility of the implants using a dental probe and listening for the muffled sound normally heard on percussion of osseointegrated implants [29].

### Micro-CT analysis

**Fig 6:**
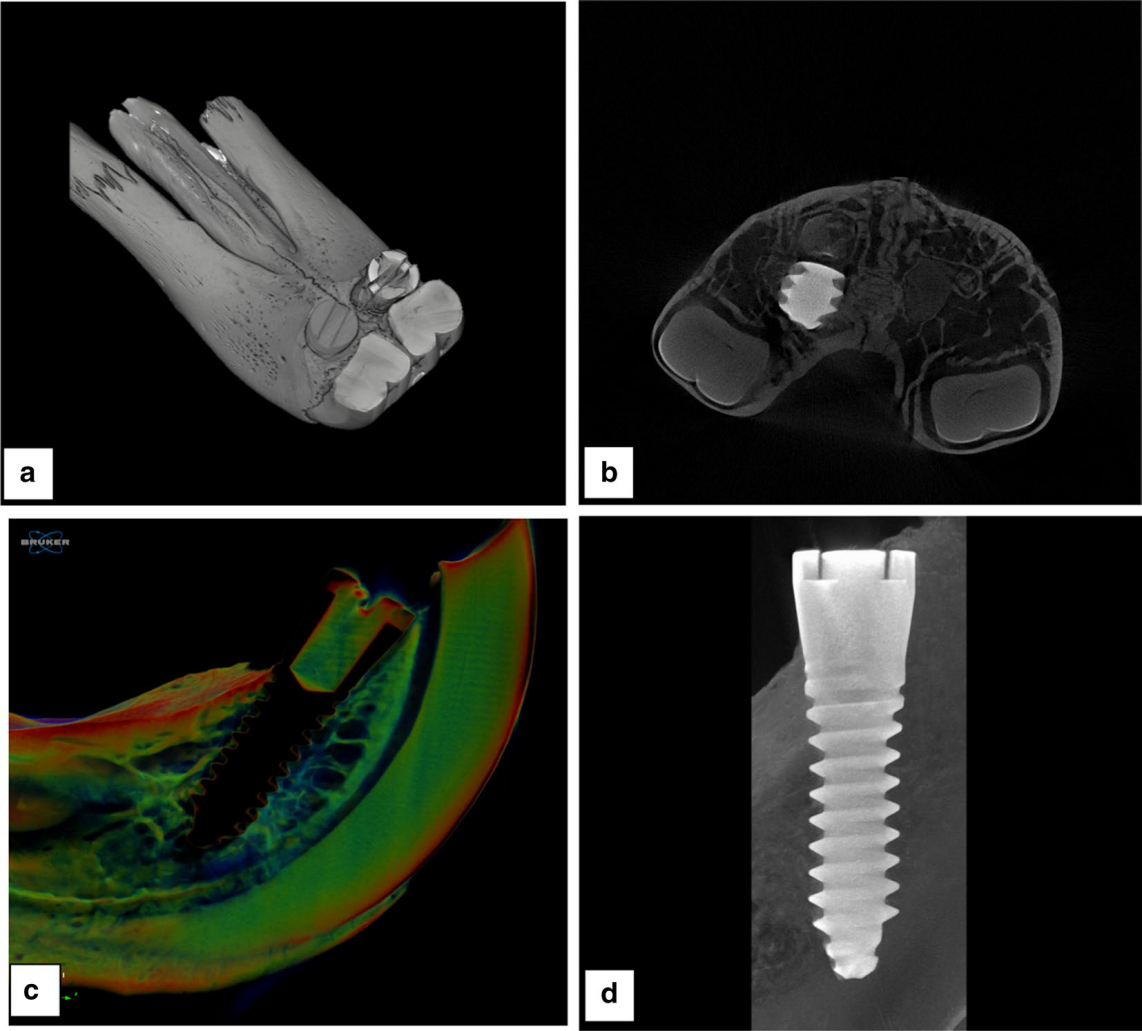
The radiographic images demonstrating the position of the implants in the presented model. **a** Three-dimensional reconstruction of the model in cadaveric rabbit using X-ray computed tomography system (XCT). **b** Representative microtomographic axial slice of the µ-CT image from rabbit maxilla. **c** Representative microtomographic sagittal slice of the µ-CT image from rabbit maxilla in colour scale. **d** Representative microtomographic coronal slice of the µ-CT image

The reconstructed 3D images of the µ-CT scans clearly demonstrated the position of the implants in the maxilla and showed close contact, with no fibrous tissue, between the implants and the surrounding bone (Fig. 6). The µ-CT captured the implants' location and allowed for the comparison of the two different implants simultaneously (Fig. 6b). The µ-CT assessments demonstrate no signs of infection or implant failure in any of the cases. In the current model, the contact between implants and surrounding bone is evaluated in three dimensions (Fig. 6b–d). Upon closer inspection, the bone tissue was on direct contact with implants' surface showing homogeneous radiodensity along the implant-tissue interface. The newly formed tissue in direct contact with the surface of the experimental implant has the same micro-structure and radiopacity to the rest of the jaw bones and similar to the bone around the control implants. Therefore, it is not unreasonable to conclude that the formed tissue on the implants' surface is bony in nature, thus confirms the osseointegration of the implants.

**Fig. 7:**
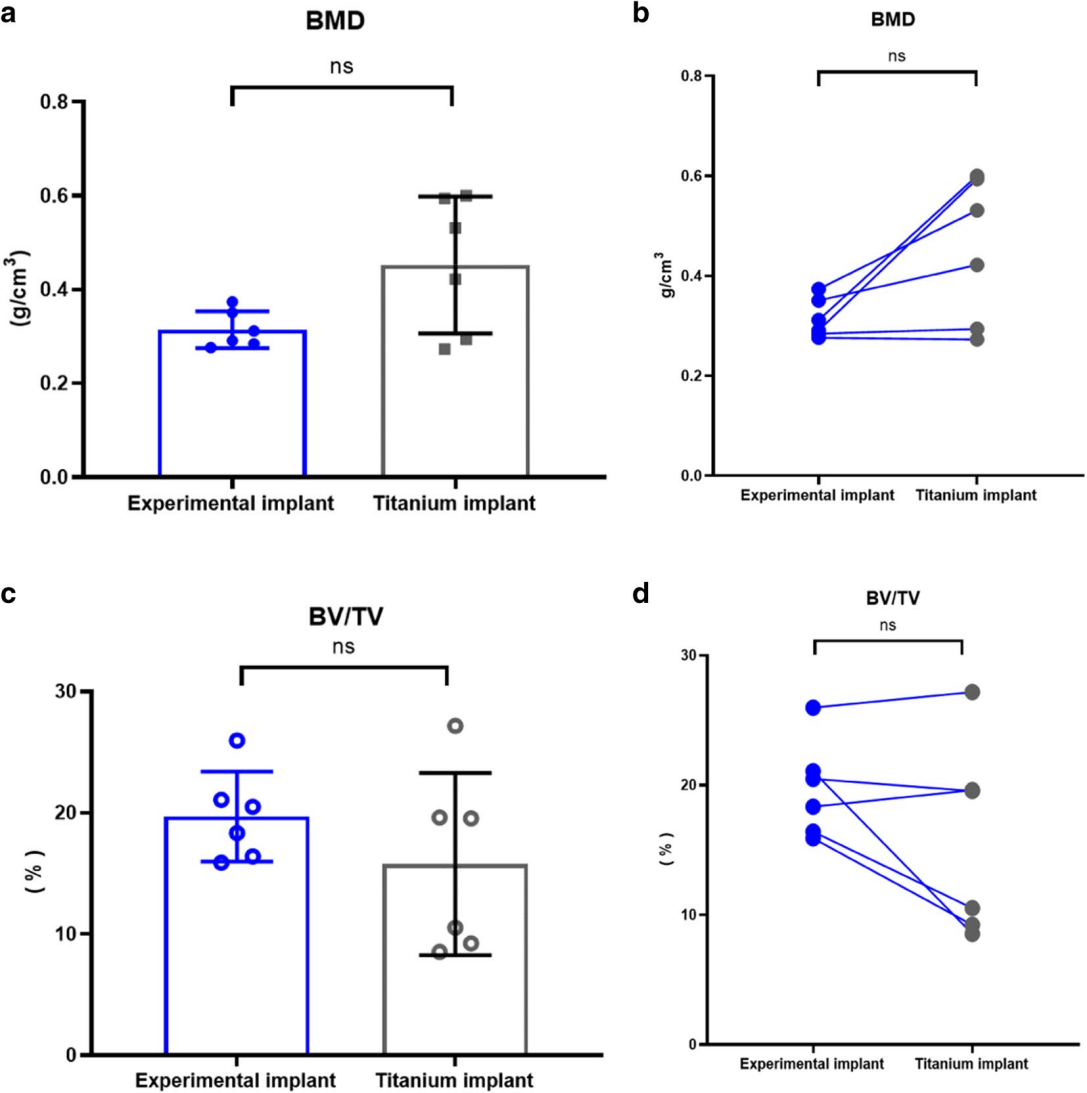
The Quantitative µ-CT analysis. **a** Bone mineral density (BMD) data are presented as mean ± SD and were analyzed with paired t-test. No statistical differences were seen between the two types of dental implants. **b** Results of paired t-tests of BMD demonstrating the pattern of the relationship between the experimental and titanium implants. The pair of implants inserted in anterior maxilla of each rabbit are connected by a line. **c** Data are presented as mean ± SD and were analyzed with paired t-test. No statistical differences were seen between the two types of dental implants. **d** Results of paired t-tests of BV/TV demonstrating the pattern of the relationship between the experimental and titanium implants. The pair of implants inserted in anterior maxilla of each rabbit are connected by a line, ns = not significant

Quantitative analysis of µ-CT data reveals that the mean bone mineral density (BMD) of the peri-implant bone was 0.32 ± 0.04 g.cm^−3^ for the experimental implants and 0.45 ± 0.15 g.cm^−3^ for the control implants (Fig. 7a, b), the difference was not statistically significant; t(5) = 2.5, (P = 0.05) with a moderate non-significant correlation coefficient between the two groups within the same animal (r = 0.39, P = 0.22). The percentage of peri-implant bone volume (BV/TV) was 19.7 ± 3.7 % for the experimental implants which is not statistically significant than that in the control group, which was 15.8 ± 7.5 %; t(5) = 1.77, (P = 0.14). A positive strong correlation was detected for bone formation related to the tested and control implants (r = 0.73, P = 0.05). There was a consistent increase of BV/TV in the experimental implant to the control implant of each rabbit (Fig. 7d). There were no statistical differences in osseointegration at 8 weeks between experimental and control implants in rabbit maxilla.

Undecalcified bone sectioning was achievable parallel to the long axis of the implants. Both implants were centralized in the bone, demonstrating direct bone tissue attached to the implants (Additional file 1: Fig. S2).

Collectively, the presented model allowed for the evaluation of the bone-implant interface in three dimensions. The three-dimensional CT of the maxilla showed the location of the two implants clearly, without overlap facilitating for the comparative analysis of different implants.

## Discussion

The primary goal of this research was to develop a reliable animal model to test the osseointegration of dental implants that overcomes the limitations of previous studies. To our knowledge, the presented model is the first to report on the application of a split-mouth design in rabbits for testing the osseointegration of dental implants in the maxilla.

Our findings provide a new perspective on the clinical simulation of the osseointegration model in *in-vivo* that is relevant to many therapeutic areas, including oral implantology, periodontology, prosthodontics, and maxillofacial surgery. In contradistinction to previous studies, our preclinical model provides a unique and reproducible preclinical setting to assess implant healing process utilizing a clinically relevant environment. The presented animal model allows the understanding of how the process of osseointegration is influenced by the environment of the oral cavity, including the presence of saliva and its associated microorganisms, during the surgery as well as the indirect forces of mastication. This better simulates the actual clinical scenario. Furthermore, it standardized the environmental and biological factors that may impact on the quality and pattern of bone regeneration around dental implants within the same animal [30, 31]. The model is also readily adaptable in medical conditions that may affect bone healing, such as diabetes and osteoporosis [32, 33]. One drawback of the current model is the fact that the implants are left submerged and not directly communicating with the oral environment. However, the submerge protocol is one of the available surgical approaches for dental implants [34]; it would be beneficial to evaluate the osseointegration in a non-submerged setting. Future studies should explore the development of abutments that would fit on these implants to allow for complete replication of the clinical picture.

All animals regained their preoperative weight within a short period of time. The surgical site was easily accessible through the conventional intraoral approach. There was minimal blood loss and low morbidity compared to other dental implant models as no extraction of primary teeth, lateralization of the inferior alveolar nerves or excessive bone removal were required [16, 35–42]. The presented model was designed considering the three fundamental principles of animal research; replacement, reduction and refinement. Also, the rabbits were considered to be a cost-effective animal model in comparison to larger animals [9].

Several animal models have been considered for the evaluation of dental implants and the testing of osseointegration, these include mice, rats, rabbits, dogs, minipigs and sheep [3, 23, 43–45]. However, in many of these models the implants were placed in extraoral locations. Dental implants have been placed in the extraction sockets of the rabbit mandibular teeth by a handful of authors [16, 37, 39–42]. Munhoz et al. in 2017 evaluated the impact of xenograft (Gen-Ox) on the osseointegration of dental implants after the extraction of the lower incisors bilaterally [16]. The removal of the lower incisors may, however, have compromised the animal welfare, the rabbits are lagomorph animals which are entirely herbivorous, and the lower incisors are crucial for chewing and biting of food [26].

Despite the close similarity, differences between the human and rabbit maxilla with regards to bone composition, mineral density and fracture toughness exist [46, 47]. Nonetheless, the intramembranous bone healing is similar in the two species [4, 14, 48]. In this model, the insertion of the dental implant in the maxilla was ideal in that it allowed for the evaluation of trabecular bone healing compared to the mandible and calvarial bones which are made up of compact bone [14]. The rabbit's maxilla has sufficient bone height and width for the placement of custom implants. The placement of implants in this anatomical region allows for the indirect mechanical loading of the tested implants from the biting forces of the lower incisors which is comparable to the clinical scenario and essential for studying osseointegration. No extraction of the primary teeth is required (only secondary incisors); therefore, the surgical trauma is minimal, and the animal welfare is not compromised. Based on the rat model used for orthopedic research, the optimum bone volume required for implant stability is 1 mm around an implant with a diameter of 2.6 mm, in tibial bone of approximately 5 mm width [18, 49]. According to the findings of the cadaveric experimentation, the available bone in the premaxilla allows placement of implants up to 8 mm in length and 2 mm diameter without damaging the adjacent primary incisors. The implant diameter for the presented model was 2 mm which is commercially available for orthodontic skeletal anchorage [50]. The results did not show bone resorption or exposure of the implant threads upon clinical and radiographical examination; this confirms that the thickness of the bone, size of the implant and socket dimension were suitable for the presented study. It has been shown that bone remodeling of the extraction socket walls during healing is essential for the initial stability of the immediate implants. However, initial bone resorption of the extraction socket during bone remodelling may cause the early loss of the dental implant [51]. Therefore more than 1 mm thickness of the bone is recommended to avoid failure of osseointegration [52–54].

The presented approach, a split-mouth design, allows two different types of dental implants to be tested simultaneously. This, eliminated anatomical variations, and avoided bias in the assessment of osseointegration and the inter-subject variability [30]. This also resulted in a reduction of the number of the rabbits needed for the study by 50% which follows the recommendations of the international guidelines for preclinical studies. Furthermore, the experimental implant can be easily randomly allocated to one side of the maxilla [30]. The accessibility of the anterior part of the maxilla facilitated the surgical procedure and reduced the realted morbidity.

The quantitative µ-CT parameters used in this study which were measures of BV/TV and BMD are indicators of the bone healing and osseointegration of dental implants. These are standard parameters used by several studies evaluating osseointegration [1, 55], or bone regeneration around the implants [56, 57]. The nature of *in-vivo* investigation mandates minimizing the number of animals as possible to draw a conclusion; however, larger numbers may prove actual differences and give more statistical power. Based on the data obtained from this pilot study, the sample size calculation can be performed for future studies.

One of the constraints for this model is the limited number of histological sections that can be obtained parallel to the longitudinal axis of the implant due to its small diameter (2 mm), this could be overcome with, multiple cross-sectional cuts. A minimum of 3–4 histological slices per implant is recommended for the 3D prediction of bone-implant contact in histological studies [58].

This model could also allow for the evaluation of osseointegration in compromised environments such as poorly controlled diabetes, osteoporosis and other immunocompromised conditions [32, 33, 59].

## Conclusions

The presented innovative model for the assessment of the osseointegration of dental implants in the rabbit maxilla is safe, reproducible, can be standardized, requires minimal surgical skills, is readily achievable with basic instrumentation and is simple to implement. The postoperative morbidity is minimal and has no associated mortality. It provides easy access to the surgical site, enables testing of the osseointegration within the maxilla and under indirect mechanical biting forces. This suggests that it may be a useful model for future preclinical implant assessment, particularly if abutments are developed to allow for direct communication of the implants with the oral environment.

## Supplementary Information

**Additional file1:** **Figure S1.** Preoperative Protocol. (a) Oral intubation and tube fixation method around the lower jaw. (b) Ophthalmic lubricant application. (c) Infraorbital nerve block. (d) The rabbit is draped in universal manner. **Figure S2.** Gross assessment of one of the explanted maxillae during preparation (cut with precision saw) for histological assessment. (a, b) The bone tissue appeared well integrated with both implants. No gap was visible between the host bone and the implants in these unstained histological sections.

## Data Availability

The datasets of this study are available from the corresponding author upon request.
