# Supplementary material for: A novel surgical model for the preclinical assessment of the osseointegration of dental implants: a surgical protocol and pilot study results
Source: J Transl Med. 2021 Jun 28;19:276. doi: 10.1186/s12967-021-02944-w (PMC8240288; doi:10.1186/s12967-021-02944-w)
Supplement: Supplementary file 1 — Additional file1: Figure S1. Preoperative Protocol. (a) Oral intubation and tube fixation method around the lower jaw. (b) Ophthalmic lubricant application. (c) Infraorbital nerve block. (d) The rabbit is draped in universal manner. Figure S2. Gross assessment of one of the explanted maxillae during preparation (cut with precision saw) for histological assessment. (a, b) The bone tissue appeared well integrated with both implants. No gap was visible between the host bone and the implants in these unstained histological sections. [file 12967_2021_2944_MOESM1_ESM.docx]

# **Supplementary information**

# **
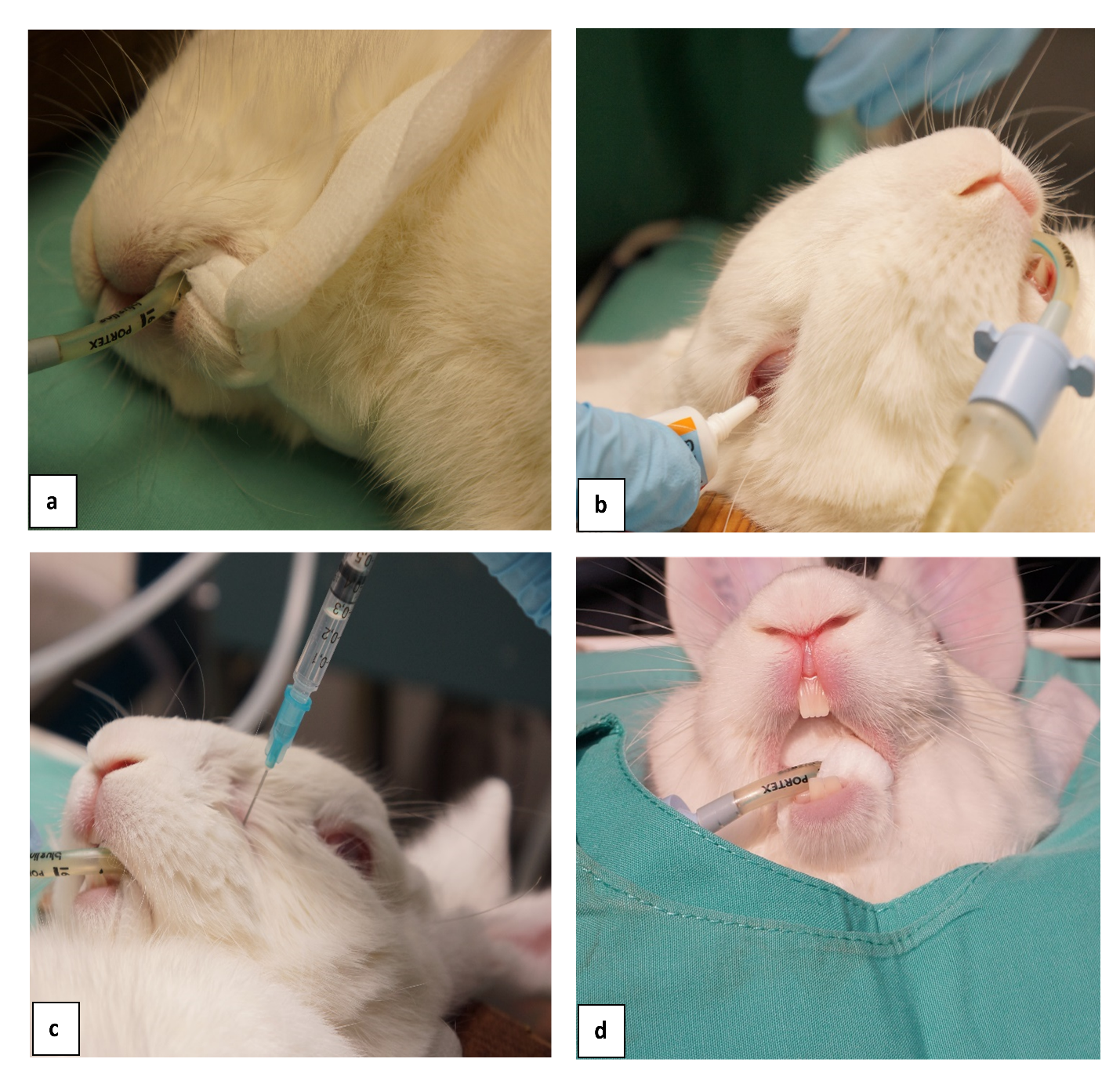
**

**Supplementary Fig.1.** **Preoperative Protocol. (a) Oral intubation and tube fixation method around the lower jaw. (b) Ophthalmic lubricant application. (c) Infraorbital nerve block. (d) The rabbit is draped in universal manner.**


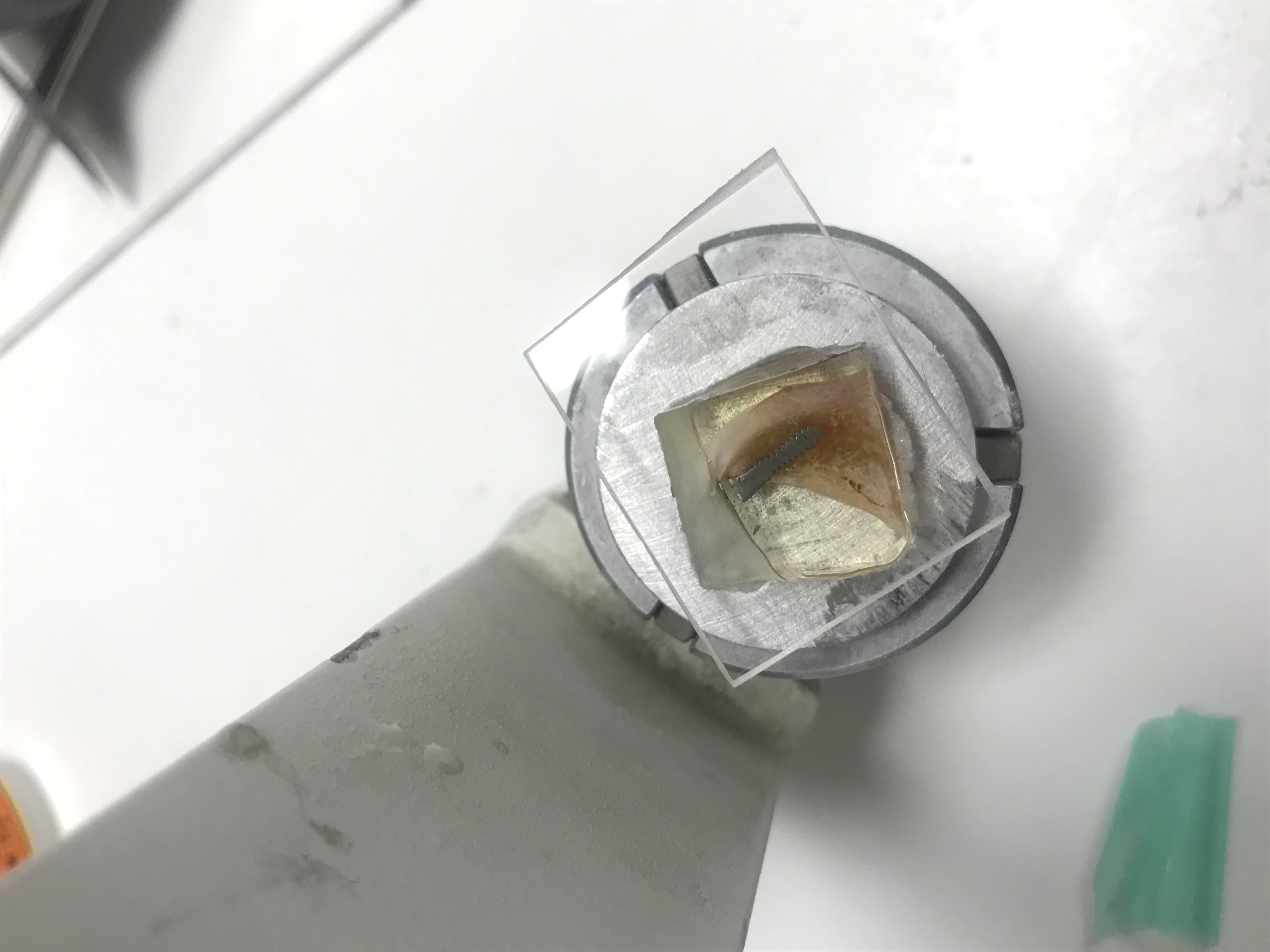

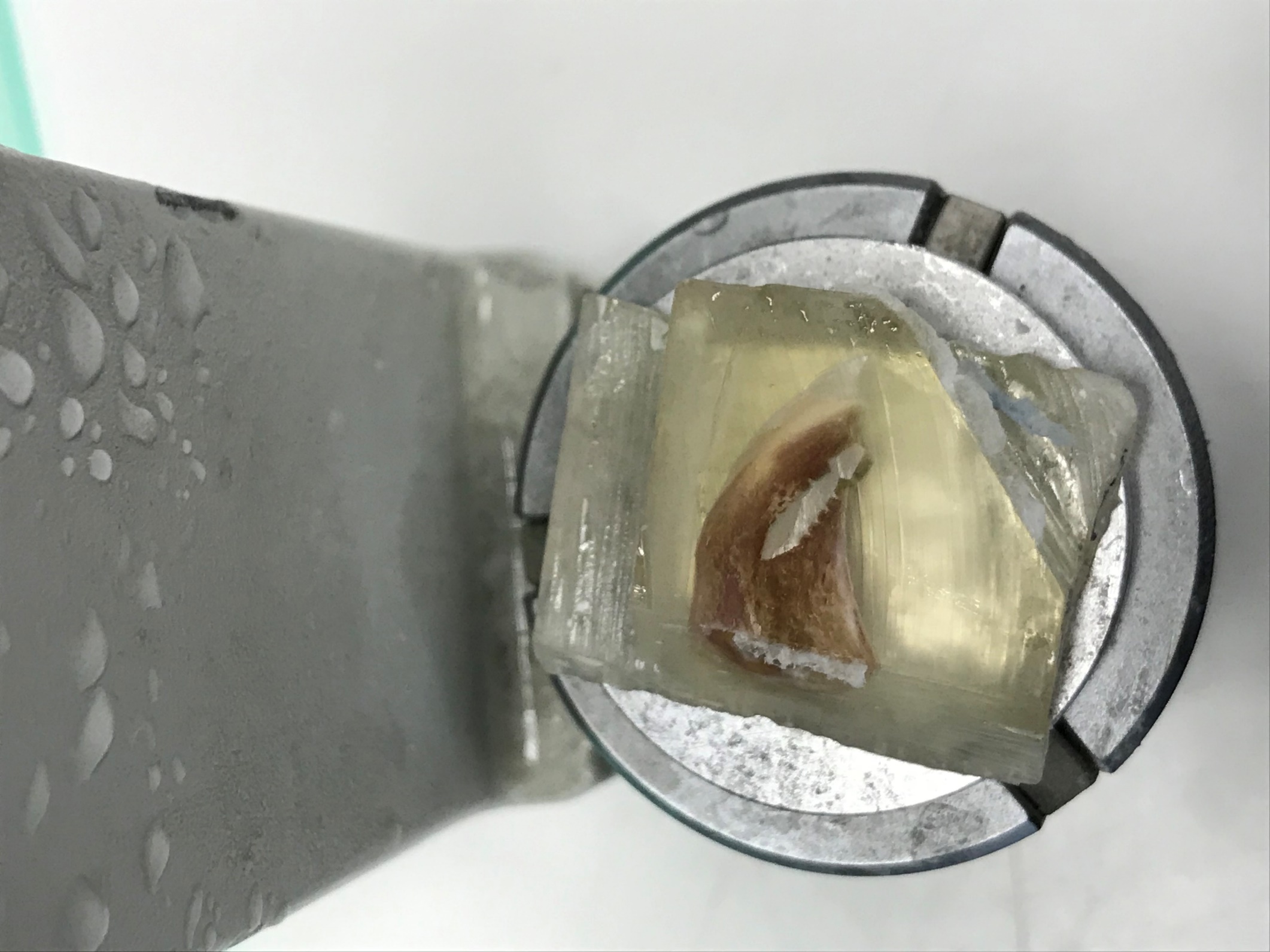


**a**

**b**

**Supplementary Fig.2. Gross assessment of one of the explanted maxillae during preparation (cut with precision saw) for histological assessment. (a & b) The bone tissue appeared well integrated with both implants. No gap was visible between the host bone and the implants in these unstained histological sections.**
